# Multidisciplinary Approach to Cover an Apex-Exposed Tooth: A Case Report after 6-Year Follow-Up

**DOI:** 10.1155/2019/8020747

**Published:** 2019-04-07

**Authors:** Jamila Kissa, Wafa El Kholti, Khadija Sekak, Sihame Chemlali

**Affiliations:** ^1^Department of Periodontology, University of Hassan II of Casablanca, Morocco; ^2^Department of Orthodontics, University of Hassan II of Casablanca, Morocco

## Abstract

**Introduction:**

The prognosis for a successful treatment of gingival recessions (GRs) is one of the main criteria for deciding whether or not and how to perform root coverage surgery. The defect-related factors are the most important to predict root coverage outcomes. Thus, severe GR could make the root coverage (RC) challenging especially in cases with advanced interdental clinical attachment loss (ICAL).

**Case Presentation:**

This case report demonstrates a challenging management of a deep localized Miller Class III GR with root apex exposure associated with ICAL. After initial therapy, the treatment had consisted of a multidisciplinary approach involving endodontic treatment, periodontal plastic surgery including a laterally positioned flap, and orthodontic treatment. The 6-year follow-up showed improvement in clinical outcomes (recession reduction (RR) and keratinized tissue (KT) augmentation) and a higher patient satisfaction.

**Conclusions:**

This case report demonstrates the role of the multidisciplinary approach in the management of deep GRs associated with ICAL. A rational choice of the RC technique was critical to achieve good clinical outcomes.

## 1. Introduction

Gingival recession (GR) can occur in subjects with either good or poor oral hygiene, and it is frequently observed in patients with destructive periodontitis [1]. In such situation, the complete root coverage may be difficult to achieve. The ultimate goal of a root coverage procedure is complete coverage of the recession defect with a good appearance related to the adjacent soft tissues and minimal probing depth following healing [1–3]. Some anatomic characteristics at baseline are considered possible predictors for root coverage outcomes, including the amount of baseline recession, the keratinized tissue (KT) around the defect, and the interdental bone level [4]. The present case report describes the decision-making process for the maintenance of a compromised tooth with poor prognosis as a result of the presence of a deep GR associated with interdental clinical attachment loss (ICAL).

## 2. Case Presentation

**Figure 1:**
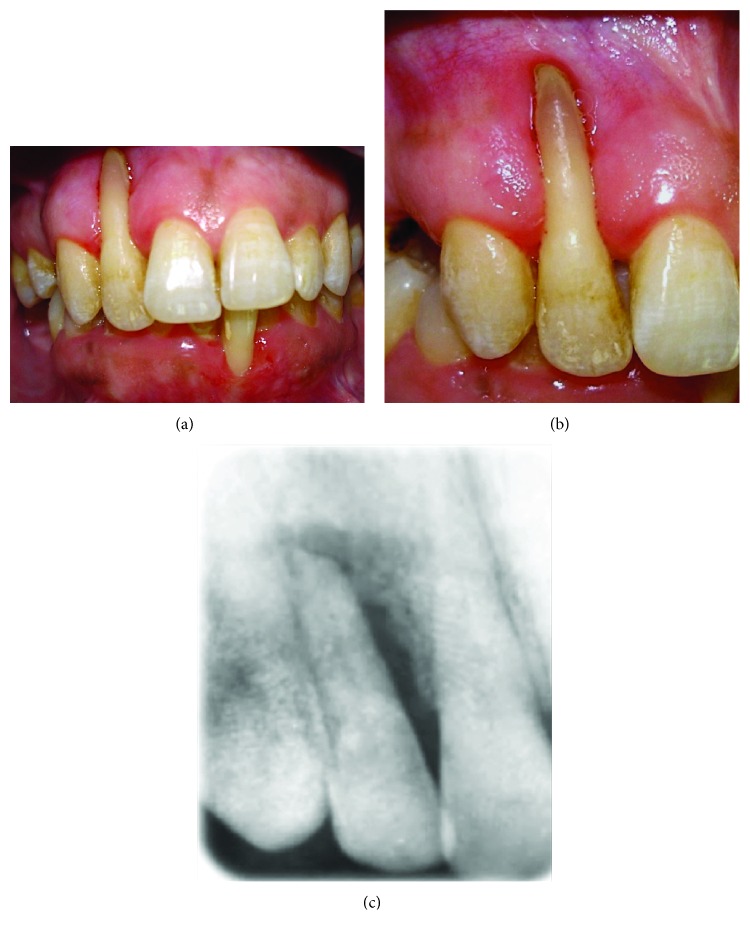
(a to c) Pretreatment clinical and radiographic images showing a deep Miller Class III GR of the tooth #2. Note a severe bone loss of the tooth and apex exposure.

**Figure 2:**
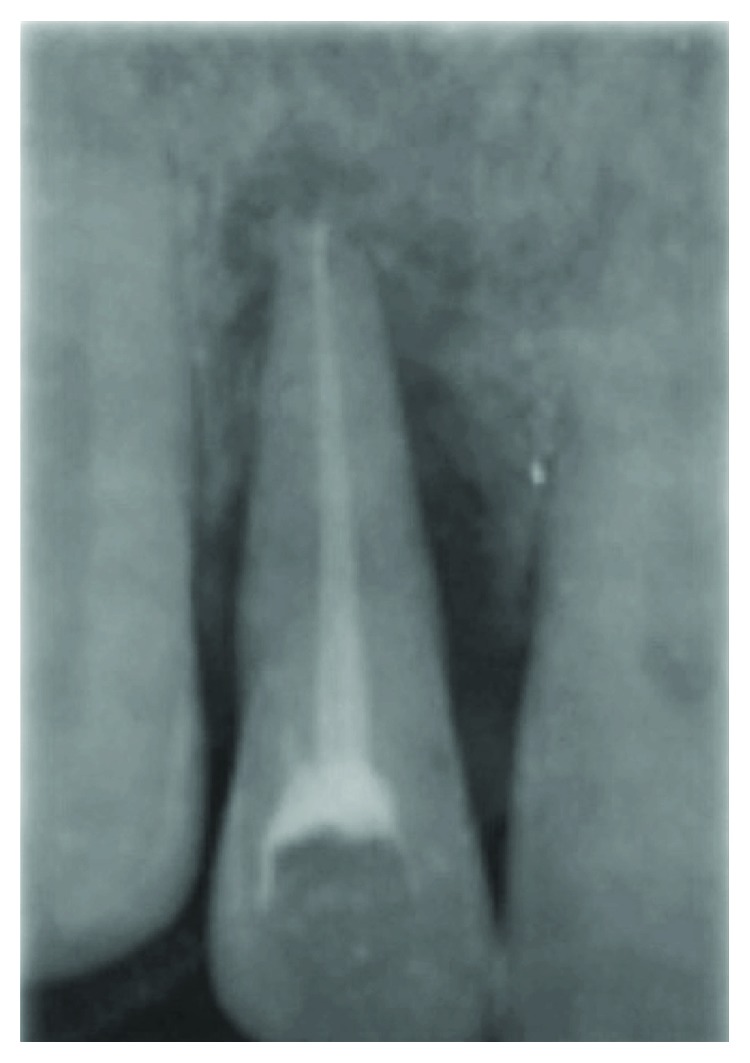
Three weeks after endodontic treatment showing osseous remineralization in the apical area.

**Figure 3:**
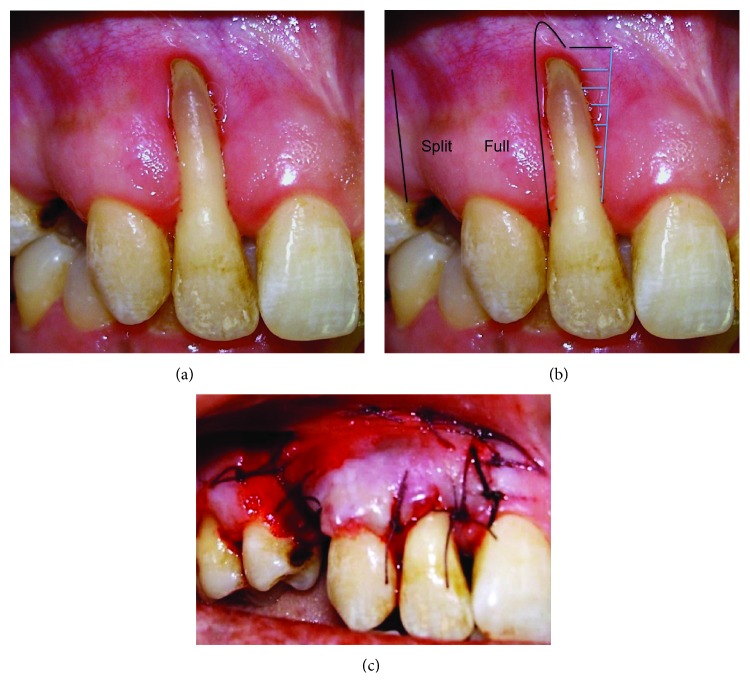
Laterally positioned flap to cover GR on the tooth #2. (a) Baseline GR. (b) Illustration of the laterally positioned flap design. Black lines represent parallel vertical incisions. Blue lines represent the marginal epithelium removed to prepare a recipient bed. (c) Postoperative view.

**Figure 4:**
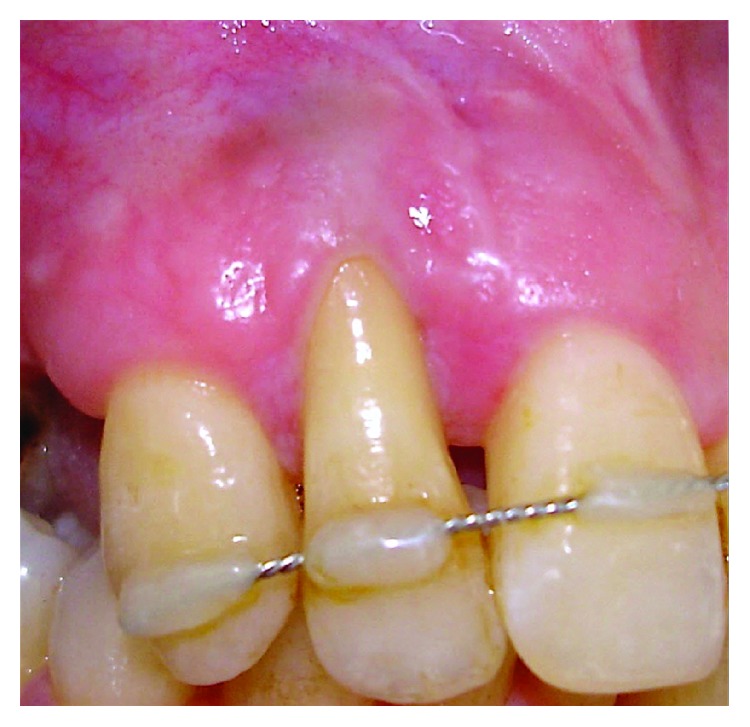
Clinical photograph at 6 months after the surgery showing a consistent reduction of baseline recession depth.

**Figure 5:**
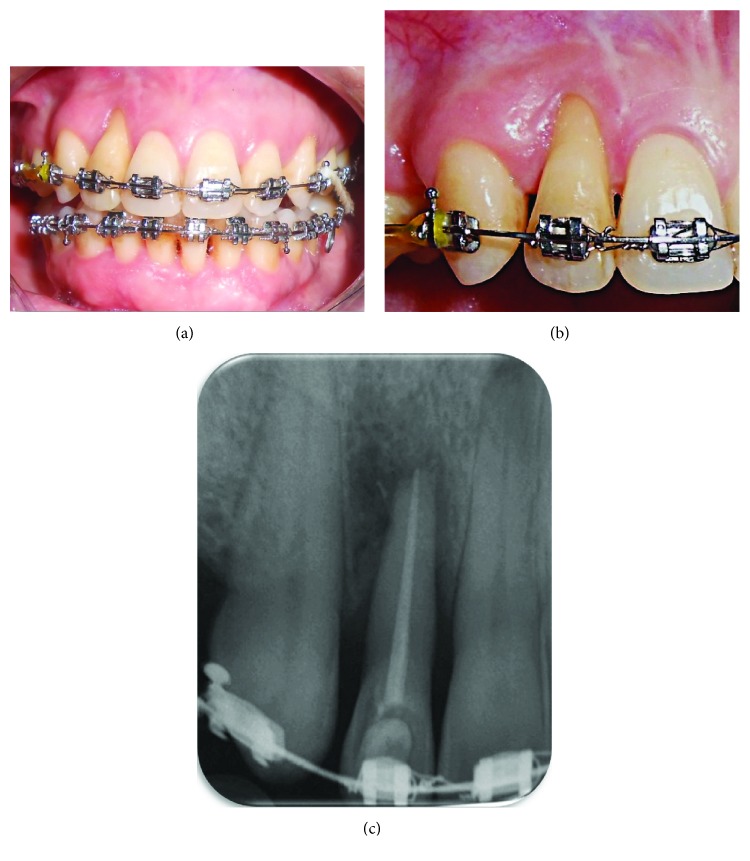
Two-year follow-up. (a, b) Facial view. Note a consistent recession reduction. (c) Periapical radiograph.

A 41-year-old, systemically healthy female presented to the Clinic of Hassan II University, Casablanca, Morocco, with esthetic complaints related to GR (Figures 1(a)–1(c)). Upon intraoral clinical examination, a deep Miller Class III GR was detected on the buccal aspect of the tooth #2. The root apex was exposed entirely and a degree 3 Muhlemann [5] mobility associated to a tooth extrusion was diagnosed. Probing examination revealed pockets of 6 mm on the buccal and palatal mesial aspect and pockets of 5 mm on the buccal and palatal distal aspect. Radiograph showed an advanced vertical bone loss on the mesial and distal aspects of the tooth with an apical lesion and mild root resorption. Thermal pulp tests indicated necrotic pulp. Occlusion was checked, and there was an occlusal trauma. A diagnosis of a periodontal-endodontic lesion in the tooth #2 was then confirmed. After oral hygiene instructions, the patient received mechanical therapy (scaling and root planing) associated with antimicrobial drugs (amoxicillin 500 mg+metronidazole 250 mg 3 times a day during 7 days). An endodontic treatment was performed on the tooth #2 (Figure 2). Two months later, based on the analysis of the possibility of tooth maintenance and on the patient's choice for a more conservative procedure, the decision was made to preserve the tooth. The aims of the corrective phase of the treatment were to cover the GR, to augment KT around the tooth #2, and to correct the occlusion by an orthodontic treatment. The chosen treatment for root coverage consisted of a lateral positioned flap (LPF) (there was a sufficient band of keratinized tissue (KT) laterally to the recession) (Figure 3(a)). Following local anesthesia, the recipient site was prepared to accommodate the LPF (Figure 3(b)). First, a V-shaped incision in the peripheral gingiva in the GR area was made followed by a wide external beveled incision on the mesial aspect and an internal beveled incision on the distal aspect creating close adaptation of the flap. An internal beveled incision toward the alveolar bone crest from the free gingival margin of the donor site was performed and continued by a distal vertical releasing incision extended to the alveolar mucosa. After that, a partial-full-thickness flap was raised. After flap incision and dissection, the exposed root surface was carefully planned with a hand curette. The prepared flap was positioned laterally to cover the GR and the removed epithelium from the mesial area of tooth #2, which was stabilized with discontinued periosteal sutures (Figure 3(c)). At 6 months, a consistent reduction of baseline recession depth (2/3 of initial baseline or 70% of root coverage) was observed (Figure 4) and an orthodontic treatment was then started in order to correct traumatic occlusion (Figures 5(a)–5(c)).

**Figure 6:**
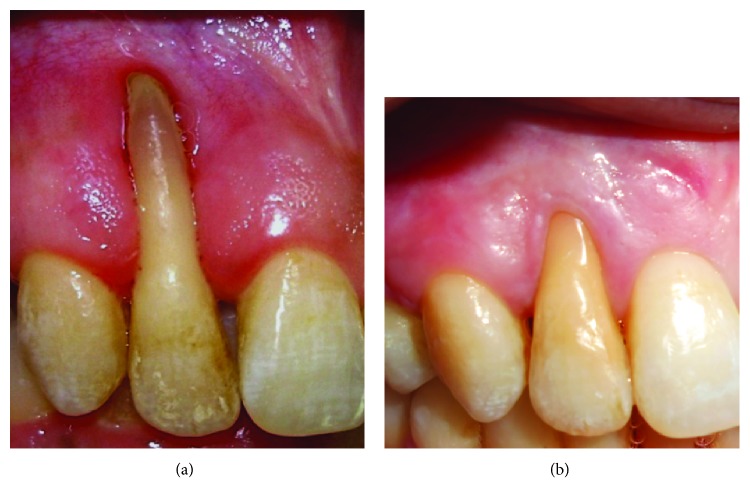
Clinical outcomes at 6 years of follow-up, at baseline, and after completion of treatment.

After 6 years of follow-up, clinically significant recession reduction (RR) (70% of root coverage), keratinized tissue (KT) augmentation (5 mm), and clinical attachment level gain were achieved. No bleeding on probing was observed during or after orthodontic treatment, and no GR was observed at the donor site (Figure 6). Regarding patient centered outcomes (recession reduction, color, and thickness of soft tissue), the patient showed a higher satisfaction.

## 3. Discussion

The indications of root coverage by surgery are mainly esthetic. Furthermore, diagnosis and elimination of the cause should be the first priority [6, 7]. After resolution of the inflammation or the elimination of trauma, the clinician may determine whether root coverage is indicated. Pathologic tooth migration (PTM) is more prevalent in teeth with more advanced clinical attachment loss [8]. If GR is associated with PTM, an orthodontic therapy should be considered. The choice of a surgical technique in periodontal plastic surgery depends on several factors that can be categorized essentially as belonging to three groups: the local anatomical characteristics of the site to be treated, the patient's requests, and the surgeon's preferences [2]. The surgical technique used in this case was the LPF. This technique has been widely used since Grupe and Warren [9] introduced this method for the treatment of deep localized GR. In this procedure, the adjacent KT is positioned laterally and the exposed root surface in the localized GR is covered. Despite its promising results on the restitution of the lost soft tissue and esthetic improvement, loss of gingiva at the donor site was frequent as well [10]. Guinard and Caffesse [11] reported an average of 1 mm of GR on the adjacent donor site. Modifications of this technique have been proposed to reduce the risk of GR at the donor site, rendering this approach highly effective. In the present case, the technique used is the method of the partial and full-thickness pedicle flap described by Ruben et al. [12]. It consists of a preparation of a full-thickness flap to cover the exposed root and a partial thickness flap near the donor site to protect the exposed root and prevent postoperative GR. After resolution of the inflammation and treatment of the periodontal-endodontic lesion, it was decided to combine the LPF with an orthodontic treatment to allow a satisfactory final esthetic result, predictable root coverage, and gain in KT. It is important to highlight that the tooth prognosis was doubtful considering the preexisting complete root exposure, advanced vertical bone loss, tooth extrusion, mobility, periodontal-endodontic lesion, and root resorption. The use of a multidisciplinary approach to save the tooth #2 had yielded to satisfactory clinical outcomes after 6 years of follow-up.
